# Macrophage potentiates the recovery of liver zonation and metabolic function after acute liver injury

**DOI:** 10.1038/s41598-021-88989-9

**Published:** 2021-05-06

**Authors:** Atsushi Miura, Takashi Hosono, Taiichiro Seki

**Affiliations:** 1 General Research Institute, Nihon University Collage of Bioresource Sciences, Fujisawa, Kanagawa 252-0880 Japan; 2 Department of Applied Life Sciences, Nihon University Graduate School of Bioresource Sciences, Fujisawa, Kanagawa 252-0880 Japan; 3 Department of Chemistry and Life Science, Nihon University Collage of Bioresource Sciences, Fujisawa, Kanagawa 252-0880 Japan

**Keywords:** Biochemistry, Biological techniques, Physiology, Biomarkers

## Abstract

The liver is an exclusive organ with tremendous regenerative capacity. Liver metabolic functions exhibit spatial heterogeneity, reflecting liver zonation. The mechanisms controlling the proliferation of hepatocytes and the accompanying matrix reconstruction during regeneration have been well explored, but the recovery potential of differentiated metabolic functions and zonation after liver injury remains unclear. We employed a mouse model of carbon tetrachloride (CCl_4_) induced-acute liver injury with clodronate-induced macrophage depletion to clarify the impact of liver injury on liver metabolism and recovery dynamics of metabolic function and liver zonation during regeneration. Depleting macrophages suppressed tissue remodelling and partially delayed cell proliferation during regeneration after liver injury. In addition, recovery of metabolic functions was delayed by suppressing the tissue remodelling caused by the depleted macrophages. The model revealed that drug metabolic function was resilient against the dysfunction caused by liver injury, but glutamine synthesis was not. Metabolomic analysis revealed that liver branched-chain amino acid (BCAA) and carbohydrate metabolism were suppressed by injury. The plasma BCAA concentration reflected recovery of hepatic function during regeneration. Our study reveals one aspect of the regenerative machinery for hepatic metabolism following acute liver injury.

## Introduction

The liver has remarkable regenerative capacity to maintain homeostasis, even after suffering severe damage^1,2^. Liver regeneration involves cell proliferation and remodelling of the tissue architecture. The liver exhibits repetitive anatomical units called liver lobules. The liver lobules are constructed with the hepatocytes arranged along a microvascular structure that distinguishes portal vein and central vein vasculature zones. In experimental models of acute liver injury, hepatocyte death in injured zones is compensated by the proliferation of adjacent hepatocytes and other hepatocytes located far away from the injured zones^3,4^. The tissue architecture is remodelled by hepatic non-parenchymal cells (NPCs)^5^. Hepatocytes dynamically remodel their metabolic functions to balance cell division and energy production during liver regeneration^6^. Such changes in liver metabolism are referred to as metabolic remodelling, and contribute to liver regeneration, cell proliferation and tissue remodelling. However, it remains unclear how tissue remodelling affects liver metabolism during regeneration, and how damaged metabolic functions regenerate after injury.

Some metabolic liver functions have spatial distribution patterns, meaning that each hepatocyte can change the expression levels of metabolic enzymes in the liver lobules. The spatial distribution patterns reflect metabolic zonation of the liver^7^. Gradients of oxygen, hormones, and nutrients in liver lobules cause spatial heterogeneity of hepatocytes. The hepatocytes located around the portal vein (periportal hepatocytes) are responsible for urea synthesis, β-oxidation, and gluconeogenesis, whereas those situated around the central vein (pericentral hepatocytes) are for glutamine synthesis, lipogenesis, and glycolysis. Metabolic zonation is compromised by liver injury^8,9^. Liver injury can affect metabolic activity; however, previous reports focused only on histological features. Changes in metabolite levels within the regenerating liver, and in drug metabolism, were not addressed.

We focused on the role of immunocytic macrophages in liver regeneration. Liver macrophages are hepatic NPCs that contribute to cell proliferation and tissue remodelling by producing pro-inflammatory cytokines and extracellular matrix (ECM) degradation enzymes^10,11^. Depleting macrophages during acute liver injury delays liver regeneration by suppressing hepatocyte proliferation and tissue remodelling^11^. Therefore, the macrophage depletion technique is suitable for investigating tissue remodelling during liver regeneration.

We investigated tissue remodelling during recovery of metabolic liver function, and its relationship with liver zonation, we hypothesised that depletion of macrophages suppresses tissue remodelling and impairs the recovery of liver metabolic functions. To address our hypothesis, we employed a mouse model of carbon tetrachloride (CCl_4_) acute liver injury with clodronate-induced macrophage depletion as a loss-of-function approach. We found that macrophage depletion impaired tissue remodelling, and partially suppressed cell proliferation. We also found that drug metabolic pathways were more resilient to injury than was glutamine synthesis. Using gas chromatography/mass spectrometry (GC/MS)- and conventional analyses, we found that branched-chain amino acid (BCAA) and carbohydrate metabolism were affected by liver injury, and that suppression of tissue remodelling did not lead to adequate recovery of these pathways during liver regeneration.

## Results

### Clodronate-liposomes delayed liver regeneration by suppressing tissue remodelling in the CCl_4_-induced acute liver injury model

**Figure 1:**
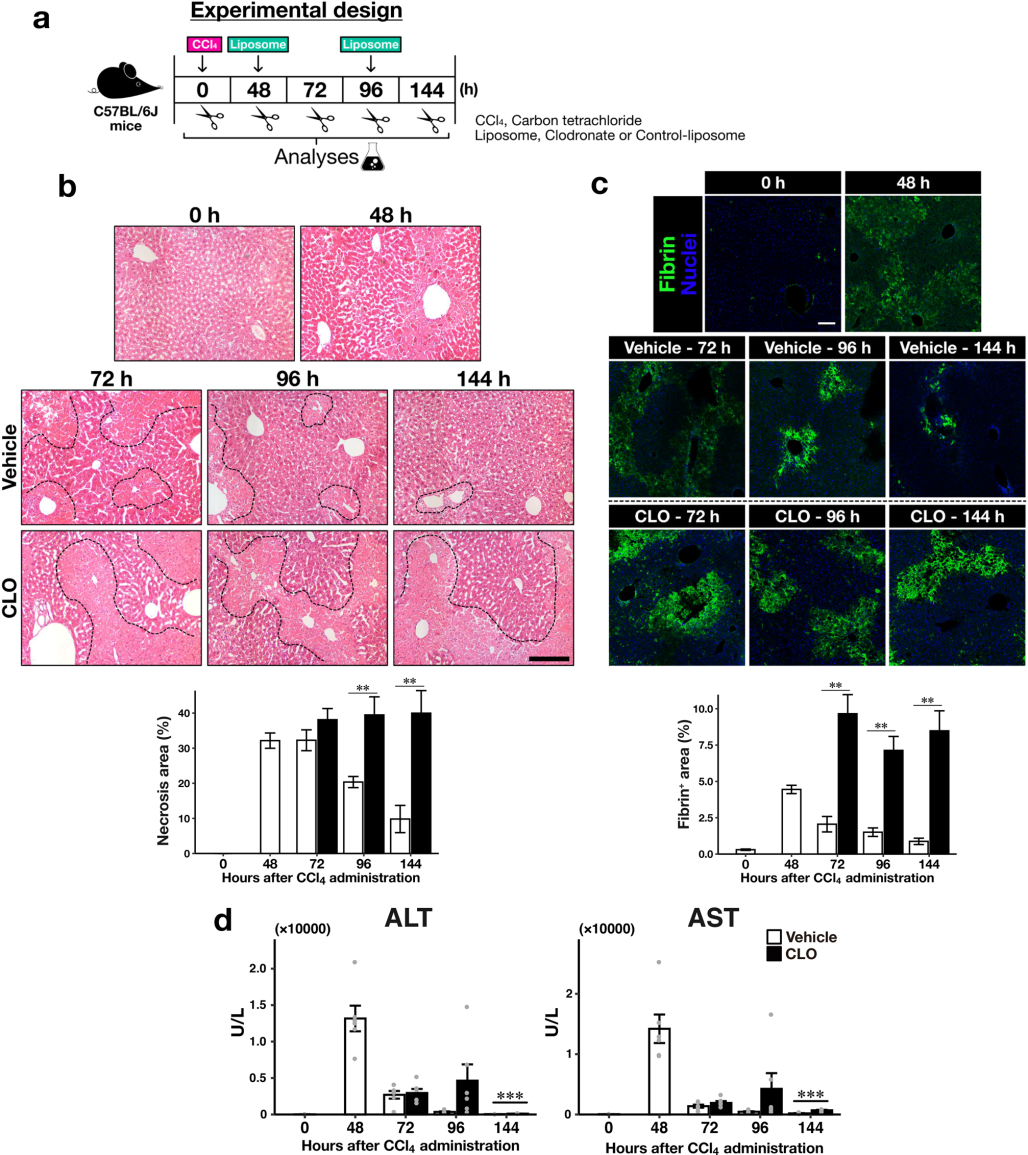
The effect of CLO treatment on CCl_4_-induced acute liver injury model mice. (**a**) Experimental design for this study. C57BL/6J mice were given CLO (100 µL/mouse, *i.p.*) 48 h after CCl_4_ (1.6 mL/kg b.w., *p.o.*). Samples were collected at 0, 48, 72, 96, and 144 h after CCl_4_ administration. (**b**) Representative images of liver sections stained with H&E (scale bar, 100 µm). Dashed lines indicate the boundaries between damaged and intact zones. (**c**) Representative images of hepatic fibrin immunofluorescent staining (scale bar, 100 µm); green, fibrin; blue, cell nuclei. (**d**) Alanine aminotransferase (ALT) and aspartate aminotransferase (AST) levels in plasma. The mean ALT level at 144 h was 45.67 ± 4.58 U/L in vehicle-treated mice and 110.83 ± 9.08 U/L in CLO-treated mice; the respective AST levels were 185.67 ± 41.33 and 698.83 ± 78.23 U/L. White bars indicate vehicle-treated mice, and black bars indicate CLO-treated mice. Data are presented as mean ± standard error of the mean (SEM; n = 6/group). Intergroup differences between the vehicle and CLO group at each time-point were compared using Welch’s *t*-test; *p < 0.05, **p < 0.01, ***p < 0.001.

We selected a pharmacological depletion technique using clodronate-loaded liposomes (CLOs) to deplete macrophages in the mouse liver. CLOs specifically induce macrophage apoptosis; thus, they have been used in experimental acute liver injury models. Administering CLOs before induction of liver injury effectively removes liver macrophages^11–14^. Depleting macrophages before induction of acute liver injury does not affect injury severity^15,16^. However, one report demonstrated that CLO pre-treatment aggravates liver injury due to enhanced inflammation^17^. Because we focused on regeneration processes and not on the initiation phase of liver injury, we injected CLOs into mice after administering CCl_4_, which induces acute liver injury, to avoid interfering with the initial inflammation (Fig. 1a). To confirm whether the CLOs depleted liver macrophages, we histologically analysed pan-macrophage marker F4/80 expression in the liver using immunofluorescent staining (Supplementary Fig. S1). F4/80-positive signals in the injured liver were decreased in CLO-treated mice at 96 and 144 h after CCl_4_ administration compared with vehicle-treated mice. Macrophages in the liver can be classified into various subsets, such as liver-resident macrophages (Kupffer cells) and monocyte-derived macrophages^18^. Thus, we measured the mRNA expression levels of typical macrophage marker genes in the liver using quantitative polymerase chain reaction (qPCR) (Supplementary Fig. S2). Gene expression levels of the pan-macrophage marker Adgre1 (F4/80 gene symbol), CD68, and the Kupffer cell marker Clec4f were reduced in the liver of CLO-treated mice compared with vehicle-treated control mice at 96 and 144 h. On the other hand, there were no changes in the mRNA expression of monocyte-derived macrophage markers Ly6c, CD11b, and Ccr2. However, the gene expression level of anti-inflammatory macrophage marker Cx3cr1 was reduced in the CLO-treated mouse liver at 144 h. These results indicate that treating mice with CLO after inducing acute liver injury depletes macrophages in the liver during liver regeneration after injury.

To assess the effect of macrophage depletion on tissue remodelling during liver regeneration, we observed the histology of the mouse liver with haematoxylin and eosin staining (Fig. 1b). Necrosis of pericentral hepatocytes is a typical histological feature of injured tissue in the CCl_4_-induced acute liver injury model. We observed severely destroyed pericentral zones at 48 h after CCl_4_ administration. The injured zones in the CLO-treated mouse livers were not repaired at 96 and 144 h compared with those in the livers of vehicle-treated mice. The blood coagulation factor fibrinogen is involved in tissue repair. Fibrin is deposited in necrotic regions of the injured liver^19^, and the deposited fibrin is subsequently cleared by macrophages via enzymatic degradation and phagocytosis^20^. We confirmed marked deposition of fibrin in pericentral zones of the injured liver at 48 h (Fig. 1c). The fibrin deposited in the injured zones remained in the CLO-treated mouse liver at 96 and 144 h. Plasma alanine aminotransferase and aspartate aminotransferase are biochemical markers of liver injury. These plasma markers were substantially higher in CLO-treated mice than those in vehicle-treated mice at 144 h (see Fig. 1d). We found that depleting macrophages during the regeneration phase suppresses tissue remodelling for injury repair.

Next, we investigated the effect of depleting macrophages on cell proliferation after liver injury. We measured cell cycle-related proteins by immunoblotting and qPCR to confirm whether depleting macrophages delayed cell proliferation (Supplementary Figs. S3 and S4). The protein expression level of proliferating cell nuclear antigen (PCNA) in the liver was not different between vehicle- and CLO-treated mice. The cyclin D1 protein level decreased slightly in the CLO-treated mouse liver at 96 h compared with that in vehicle-treated mice (Supplementary Fig. S3). The expression level of Ccnd1 mRNA, which is the gene encoding the cyclin D1 protein, decreased slightly but not significantly in the CLO-treated mouse liver at 96 h (Supplementary Fig. S4). The gene expression levels of Ccna2 and Ccnb1 in the CLO-treated mouse liver were decreased at 96 h, whereas Ccne1 expression was comparable between vehicle- and CLO-treated mice. We histologically analysed the proliferative marker Ki-67, which is highly expressed during S phase of the cell cycle^21^, using immunohistochemistry (Supplementary Fig. S5). The percentage of Ki-67-positive nuclei in the liver of CLO-treated mice was lower than that in vehicle-treated mice at 72 h. The percentage of Ki-67 positive nuclei in the CLO-treated mouse liver was increased at 96 and 144 h. Based on these findings, we conclude that depleting macrophages partially delayed cell proliferation during regeneration. Because macrophage primes hepatocyte proliferation during liver regeneration through the production of inflammatory cytokines such as tumor necrosis factor-α (TNF-α) and interleukin-6 (IL-6)^11^, we further analysed the gene expression of Tnfa and Il6. Macrophage depletion did not affect the expression levels of these genes (Supplementary Fig. S6). Also, to confirm whether the macrophage depletion induces apoptotic cell death during liver regeneration, we histologically detected apoptotic cells in the liver (Supplementary Fig. S7). The number of apoptotic cells in the CLO-treated mouse liver was higher than that in vehicle-treated mouse liver at 96 h. These results suggest that CLO-treatment induces apoptotic cell death in the liver during the regeneration phase. Taken together, our experimental mouse model revealed suppressed tissue remodelling and delayed cell proliferation during the regenerative process after acute liver injury.

### Suppression of tissue remodelling during regeneration did not affect the recovery of hepatic drug metabolism

**Figure 2:**
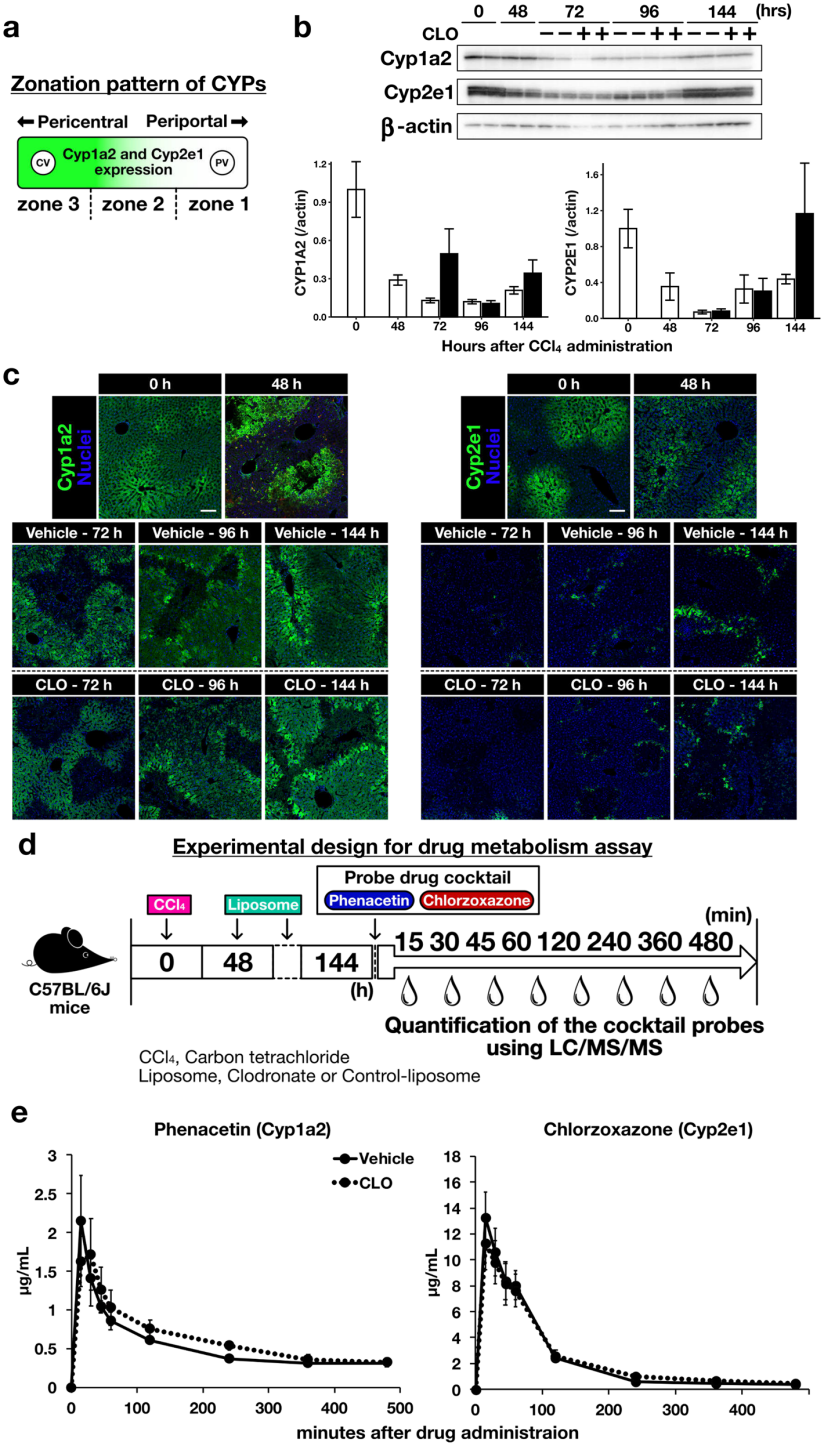
Effect of tissue remodelling suppression on drug metabolism during regeneration. (**a**) Zonation of cytochrome P450 enzymes (CYPs) in liver lobules. CYP expression by hepatocytes decreased gradually toward the periportal zones. Hepatocytes located in zone 1 of a healthy liver did not express CYPs. CV, central vein; PV, portal vein. (**b**) Cyp1a2 and Cyp2e1 protein expression levels measured by immunoblotting. β-actin was used as the loading control. (**c**) Representative immunofluorescent staining images of Cyp1a2 and Cyp2e1 (scale bar, 100 µm). Green, Cyp1a2 and Cyp2e1; blue, cell nuclei. (**d**) Experimental assessment of drug-metabolising capacity. C57BL/6 J mice were given CLO (100 µL/mouse, *i.p.*) 48 h after CCl_4_ (1.6 mL/kg b.w., *p.o.*). These mice also received the probe drugs phenacetin and chlorzoxazone 144 h after CCl_4_ administration. Blood samples were collected at 15, 30, 45, 60, 120, 240, 360, and 480 min after administration of the probes. The drug concentrations in plasma were quantified by LC/MS/MS. (**e**) Plasma concentration–time curves of phenacetin and chlorzoxazone. Solid lines denote vehicle-treated mice and dashed lines CLO-treated mice. Data are presented as mean ± SEM (n = 6/group).

The liver, which is zonated, plays a major role in drug metabolism^7^. We investigated the recovery of drug metabolism during liver regeneration in the context of liver zonation. CCl_4_ is metabolised to a trichloromethyl free radical by cytochrome P450 Cyp2e1. Radical peroxidation of hepatocyte lipids triggers necrotic cell death^22^. Cyp2e1 is expressed by pericentral hepatocytes^23^. CCl_4_ destroys the pericentral zones of liver lobules, leading to dysfunctional drug metabolism. To assess the effect of tissue remodelling suppression on the recovery of drug metabolism and zonation patterns during liver regeneration, we measured the levels of cytochrome P450 enzymes (CYPs) in the pericentral zone. Unlike Cyp2e1, Cyp1a2 was distributed in the pericentral region (Fig. 2a). We measured liver Cyp1a2 and Cyp2e1 protein levels via immunoblotting (Fig. 2b). Cyp1a2 levels decreased at 72 and 96 h after CCl_4_ administration. Cyp2e1 levels decreased from 48 to 96 h; there were no differences between the between vehicle- and CLO-treated mice. Immunofluorescent staining showed the histological distribution of Cyp1a2 and Cyp2e1 in the liver (Fig. 2c). CYP metabolism was severely compromised by liver injury in both groups, and did not fully recover during the observation period. Cyp1a2- and Cyp2e1-expressing hepatocytes were adjacent to damaged pericentral zones in the injured liver. However, we found no differences in expression between vehicle- and CLO-treated mice at 144 h after CCl_4_ administration.

To confirm functional recovery of drug metabolism in vivo, we analysed the metabolism of drug probes by the regenerating liver (Fig. 2d). Phenacetin and chlorzoxazone, (CYP substrates) were used to analyse Cyp1a2 and Cyp2e1 activity, respectively. The blood concentrations of these drugs in CLO-treated mice were comparable to those in vehicle-treated mice at 144 h after CCl_4_ administration (Fig. 2e). The mean area under the concentration–time curve (AUC) of phenacetin was 425 ± 28.6 µg min/mL in vehicle-treated mice and 436 ± 45.1 µg min/mL in CLO-treated mice (*p* = 0.627). For chlorzoxazone, the respective values were 1150 ± 22.4 and 1170 ± 28.4 µg min/mL (*p* = 0.207). Thus, suppression of tissue remodelling during liver regeneration did not affect the recovery of drug metabolism.

### Suppression of tissue remodelling partially compromised the recovery of nutrient metabolism

**Figure 3:**
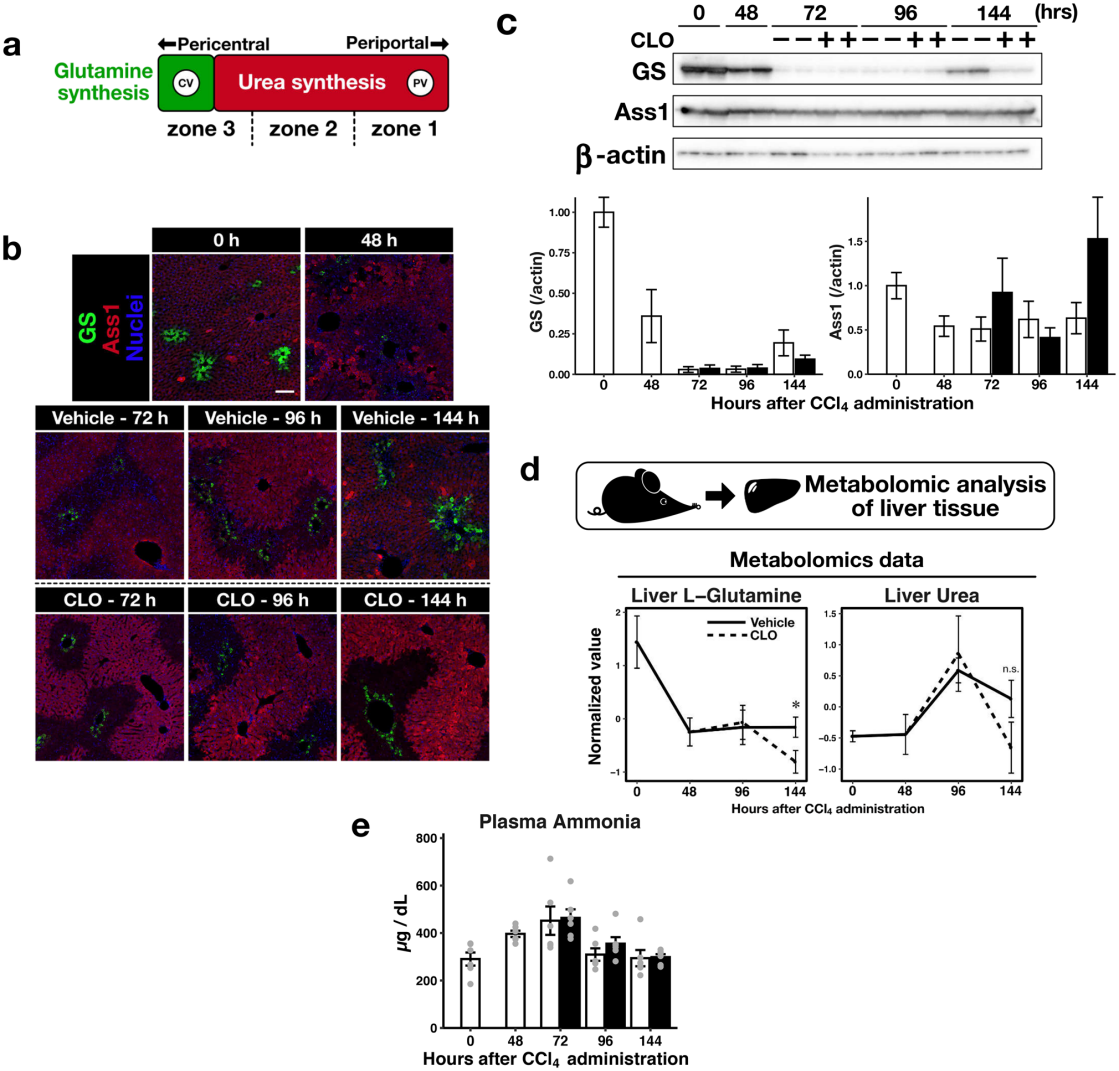
Effects of tissue remodelling suppression on glutamine and urea synthesis during regeneration. (**a**) Zonation patterns of glutamine and urea synthesis in liver lobules. Glutamine was synthesised by hepatocytes surrounding the central veins. Urea was synthesised by all hepatocytes except those surrounding the central vein. *CV* central vein; *PV* portal vein. (**b**) Representative immunofluorescence staining images of glutamine synthetase (GS) and argininosuccinate synthase 1 (Ass1) (scale bar, 100 µm); green, GS; red, Ass1; blue, cell nuclei. (**c**) GS and Ass1 protein expression levels measured by immunoblotting. β-actin was used as the loading control. (**d**) Liver glutamine and urea levels revealed by metabolomic analysis. (**e**) Plasma ammonia levels. Data are presented as mean ± SEM (n = 6/group). The vehicle and CLO groups were compared at each time point using Welch’s *t*-test; *p < 0.05.

The metabolic pathways of liver nutrients, including glutamine and urea, are zonated^7^. We explored whether suppression of tissue remodelling affected the recovery of these metabolic functions, and the zonation patterns. Glutamine synthetase (GS), which converts glutamate to glutamine, is expressed only by pericentral hepatocytes. In contrast, argininosuccinate synthase 1 (Ass1), which is an enzyme involved in urea synthesis, is expressed by all hepatocytes except pericentral hepatocytes^23^ (Fig. 3a). We analysed the distribution patterns of GS and Ass1 during liver regeneration using immunofluorescent staining (Fig. 3b). The zonation patterns of GS and Ass1 were clearly disturbed 48 h after CCl_4_ administration, and recovered only incompletely in CLO-treated mouse livers compared to vehicle-treated livers at 144 h. We measured GS and Ass1 expression levels via immunoblotting (Fig. 3c). The GS level was remarkably reduced between 48 and 96 h after CCl_4_ administration. Moreover, GS expression was suppressed 144 h after CLO treatment. In contrast, Ass1 expression was not affected by liver injury or CLO treatment. GC/MS was used to obtain metabolomic data. The glutamine level in the liver of CLO-treated mice was lower than that of vehicle-treated mice at 144 h (Fig. 3d), but the hepatic urea level did not decrease after CLO treatment. GS regulates ammonia detoxification; thus, we also analysed the plasma ammonia levels in these mice. The plasma ammonia levels were comparable between the vehicle-treated mice and the CLO-treated mice (Fig. 3e). Together, these results indicate that suppression of tissue remodelling delayed functional recovery of nutrient metabolism during regeneration after liver injury, and affected zonation patterns.

### Suppressed tissue remodelling causes insufficient recovery of BCAAs and carbohydrate metabolism

**Figure 4:**
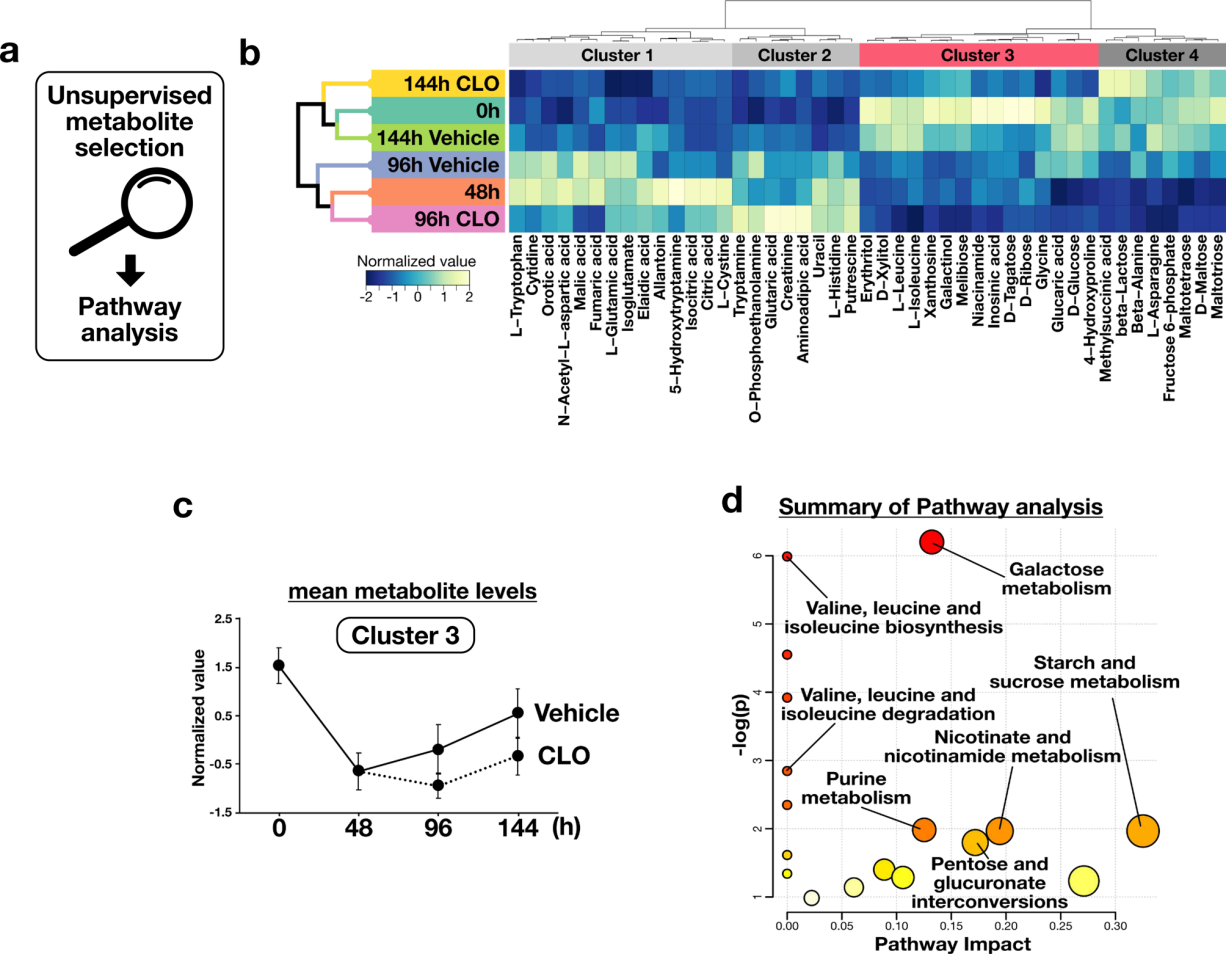
Characterisation of the liver metabolome during regeneration after acute liver injury. (**a**) Characterisation of the liver metabolome by GC/MS. Bioinformatic data were collected, and multivariate analyses were performed by using R software. (**b**) Heatmap of selected metabolites. The coloured blocks indicate group mean levels. Hierarchical clustering was performed using the correlation-based distance and the ward.D2 clustering method. (**c**) Group mean levels of metabolites classified in Cluster 3. (**d**) The results of pathway analysis performed using Metaboanalyst 4.0. The group mean levels are presented as mean ± standard deviation (SD; 15 metabolites in each group).

As suppression of tissue remodelling affected the functional recovery of nutrient metabolism, we used unsupervised multivariate analyses to further explore potential metabolic pathways closely related to liver injury (Fig. 4a). We hypothesised that transiently decreased metabolites during the regeneration phase reflect recovery of liver metabolic functions; thus, we tried to identify metabolites that changed over time in the vehicle-treated mouse liver using principal component analysis (PCA) (Supplementary Fig. S8). The PCA score plot showed that the mouse liver metabolite profile changed parallel with the PC1 axis at each time point. Therefore, the PC1 axis indicated a lapse of time after liver injury, so we performed Pearson’s correlation analysis between the PC1 score and the normalised peak intensity of every metabolite. We selected metabolites based on statistical criteria set at a false discovery rate < 0.05 with Pearson’s correlation coefficient more/less than 0.5. To identify differences in metabolites between vehicle- and CLO-treated mice, we clustered the selected metabolites in all mouse liver groups using hierarchical clustering (Fig. 4b). We divided the metabolites into four clusters based on the group differences, and determined that Cluster 3 included metabolites that transiently decreased after liver injury. Notably, the metabolite levels of the CLO-treated mouse liver in Cluster 3 were decreased compared with those in the vehicle-treated mouse liver at 96 and 144 h after CCl_4_ administration (Fig. 4c). We performed an over-representation pathway analysis to identify the metabolic pathways affected by liver injury. This analysis functionally categorised metabolites into biological pathways (Fig. 4d). The results of the pathway analysis showed that the BCAA and carbohydrate metabolic pathways, such as galactose and starch-sucrose metabolism, were suppressed during the regeneration phase after liver injury. Additionally, recovery of these pathways was delayed due to suppressed tissue remodelling during regeneration.

**Figure 5:**
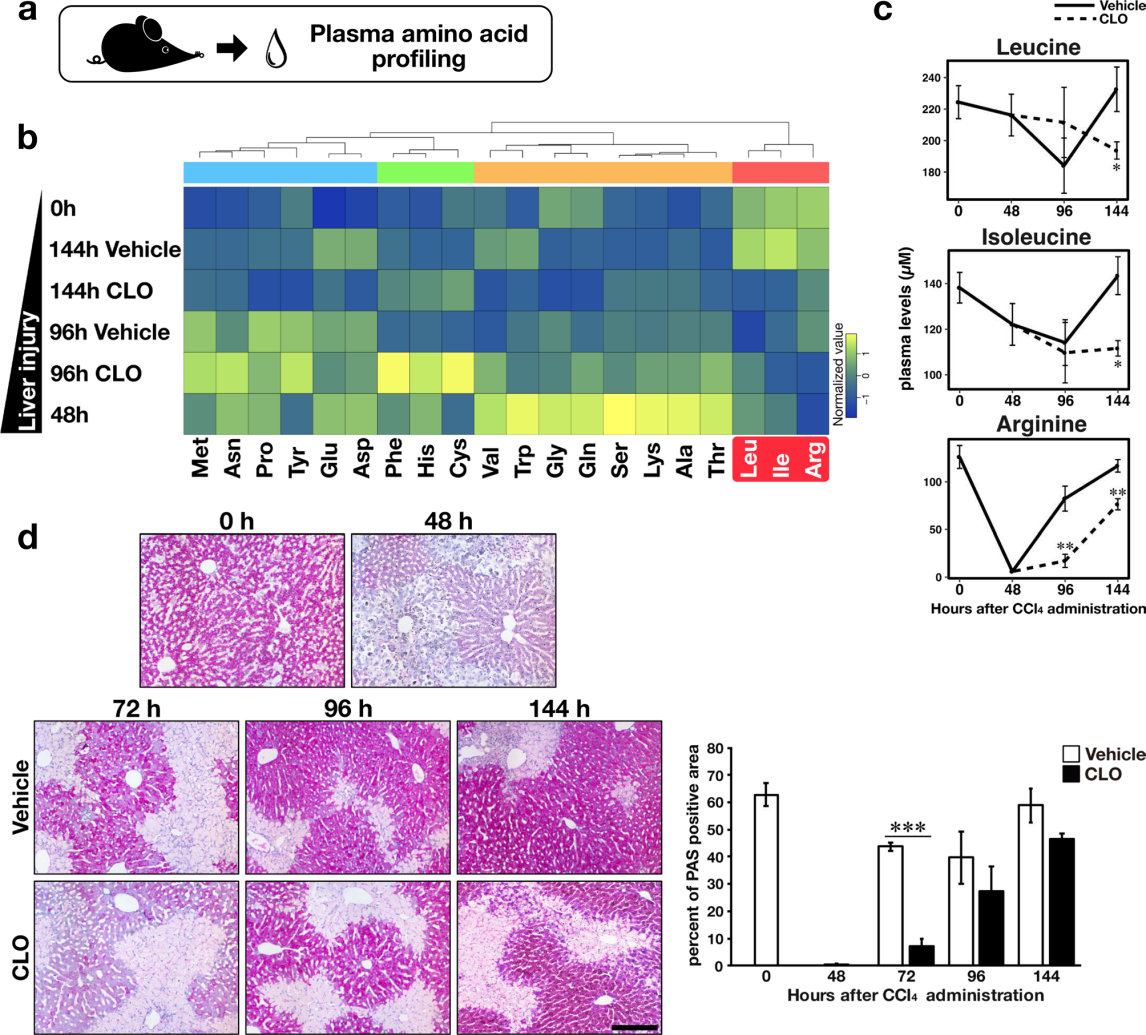
The effects of tissue remodelling suppression on the recovery of amino acid and carbohydrate metabolism during regeneration. (**a**) Plasma amino acid levels were quantified by LC/MS/MS. Changes in plasma amino acids levels are shown as a heatmap (**b**). The coloured blocks indicate group mean levels. Hierarchical clustering was performed using the correlation-based distance with the ward.D2 clustering method. (**c**) Plasma levels of leucine, isoleucine, and arginine. (**d**) Representative Periodic acid Schiff (PAS) stained images (scale bar, 100 µm). Intracellular glycogen is stained purple. Images were quantified as follows: purple dye-stained areas were divided by total tissue areas of the same images. White bars indicate vehicle-treated mice and black bars indicate CLO-treated mice. Data are presented as mean ± SEM (n = 6/group). Intergroup differences between the vehicle and CLO group at each time-point were compared using Welch’s *t*-test; *p < 0.05, **p < 0.01, ***p < 0.001.

We measured free amino acids in blood plasma to verify whether suppressing tissue remodelling delays the recovery of BCAA metabolism during regeneration after liver injury (Fig. 5a). The results of plasma amino acid profiling showed that BCAA, leucine, isoleucine, and the basic amino acid arginine were decreased in the injured mouse liver (Fig. 5b). As shown in Fig. 5c, the plasma levels of these amino acids were lower in CLO-treated mice than those in vehicle-treated mice at 144 h after CCl_4_ administration.

To assess the effect of suppressing tissue remodelling on the recovery of carbohydrate metabolism during liver regeneration, we reanalysed the metabolomics data in terms of carbohydrate metabolism. The hepatic glucose level in CLO-treated mice tended to decrease compared with that in the vehicle-treated mice at 96 h after CCl_4_ administration (Supplementary Fig. S9). Glucose is stored as glycogen in the liver and muscle, and the blood glucose level is maintained through the breakdown of liver glycogen; therefore, we histologically confirmed glycogen storage in the liver (Fig. 5d). The histological evaluation of glycogen with Periodic acid Schiff (PAS) staining indicated that glycogen storage capacity was decreased markedly in the injured liver at 48 h after CCl_4_ administration. The percentage of PAS-stained area in the livers of CLO-treated mice was considerably lower than that of vehicle-treated mice at 72 h. In addition, the PAS stained area in the CLO-treated mouse liver tended to decrease compared with the vehicle-treated mouse liver at 96 and 144 h.

Taken together, these findings demonstrate that BCAA and carbohydrate metabolism was suppressed by liver injury, and insufficient tissue remodelling delayed the recovery of these metabolic pathways.

## Discussion

Suppression of tissue remodelling delayed the recovery of GS expression and liver glutamine levels after injury. However, neither the hepatic Ass1 expression level nor the urea level was affected (Fig. 3), in agreement with a previous report showing that glutamine, but not urea, synthesis was suppressed by CCl_4_-induced acute liver injury^24^. Unexpectedly, suppression of tissue remodelling did not affect the recovery of drug metabolism (Fig. 2). This is explained by the different zonation patterns of GS and CYPs. GS is expressed by hepatocytes located around the central vein. In contrast, Cyp1a2 and Cyp2e1 are expressed by hepatocytes located in the pericentral and middle zones of the liver lobules^23^. Cyp2e1 expression increases locally in hepatocytes adjacent to the injured pericentral zone during regeneration in the CCl_4_-induced acute liver injury model^8^. In addition, Cyp2e1-expressing hepatocytes located in the middle zone are resistant to liver injury, whereas GS-expressing hepatocytes are destroyed by CCl_4_^9^. Consistent with these observations, Cyp2e1 and Cyp1a2 were locally expressed in hepatocytes surrounding injured liver zones (Fig. 2c). However, GS expression was not completely abolished after liver injury (Fig. 3b). The reason why the GS expression pattern differed between our study and other reports is unclear, although our findings highlight the impact of liver injury on hepatic metabolic function, which is governed by the zonation patterns of metabolic enzymes expressed by hepatocytes. Ma et al. also found that re-establishment of the zonation of Cyp2e1 and GS expression differed in the regenerating liver after acute injury^9^. Therefore, differences in drug metabolism and glutamine synthesis may not only reflect the degree of susceptibility to liver injury, but also the mechanisms by which zonation patterns are re-established.

Schenk et al. used pharmacokinetic modelling to study the relationship between CYP zonation patterns and in vivo activity. CYP activity decreased when hepatocytes were lost to injury soon after CCl_4_ administration^25^. The liver drug-metabolising capacity decreased because the surviving hepatocytes were dysfunctional, although they continued to express CYP. In contrast, we clearly showed that the hepatocytes of injured zones expressed Cyp1a2 and Cyp2e1 during regeneration, compensating for local dysfunction caused by the death of other hepatocytes even when tissue remodelling was suppressed. Hence, the drug-metabolising capacity of the liver is maintained during regeneration after acute liver injury. The compensatory mechanisms remain unknown; further research is required.

Plasma amino acids have been investigated as potential biomarkers to predict the severity of non-alcoholic fatty liver disease^26^ and the mortality of end-stage liver diseases^27^. Holecek et al. reported that the plasma BCAA levels of CCl_4_-injured rats are lower than those of intact rats during the regeneration phase after acute liver injury^28^. We demonstrated that suppressing tissue remodelling decreased plasma levels of leucine and isoleucine during the regeneration phase (Fig. 5c). Thus, our results highlight that plasma BCAA levels reflect the recovery of hepatic function during regeneration. Moreover, plasma arginine has been identified as a specific biomarker of acute liver injury^29^, which was also confirmed in the current study (Fig. 5c). Taken together, we propose that plasma BCAAs and arginine are biomarkers for liver regeneration after acute liver injury.

BCAAs not only play a role as a biomarker in liver diseases but are bioactive substances in the liver. The mammalian target of rapamycin (mTOR) signalling pathway, which is activated by BCAAs^30^, promotes proliferation of hepatocytes during liver regeneration^31^. Thus, BCAAs contribute to hepatocyte proliferation during liver regeneration. Indeed, supplementing with BCAAs has been shown to promote liver regeneration in several animal models^32,33^. In this study, intra-hepatic levels of leucine and isoleucine were decreased in CLO-treated mice at 144 h after CCl_4_ administration (Fig. 4b). Also, the number of proliferating hepatic cells in CLO-treated mice was higher than that of vehicle-treated mice at 144 h (Fig. S5), which is the same time point as the reductions in hepatic leucine/isoleucine (Fig. 4b). These findings indicate that the reductions in hepatic BCAAs and upregulation of cell proliferation occurred simultaneously, which conflicts with previous evidence that administering BCAAs promotes liver regeneration. Why did hepatic BCAAs decrease during the regeneration phase in this study? The liver mainly uses BCAAs for protein synthesis, because the liver has quite low BCAA catabolic activity for energy production^34^. In addition, protein synthesis is upregulated during liver regeneration^35^. Thus, we conclude that hepatic cells consume BCAAs as protein building blocks for cell proliferation during liver regeneration, which may be why hepatic BCAAs decreased during the regeneration phase of this study.

Systemic energy expenditure increases, whereas energy production from carbohydrates decreases, in patients with acute liver failure^36^. Rat liver perfusion experiments have demonstrated that liver injury suppresses the incorporation of hepatic glucose^37^. In addition, supplementing with glucose delays liver regeneration by suppressing cell proliferation in a partial hepatectomy model of mice^38^. Also, proliferating hepatocytes have little stored glycogen, whereas neighbouring quiescent hepatocytes increase storage of glycogen during liver regeneration^39^. These findings suggest that reduced carbohydrate (or glucose) utilisation is linked with the proliferation potential of hepatocytes for regeneration. In this study, liver glucose and glycogen levels decreased transiently after liver injury, and these levels decreased persistently by suppressing tissue remodelling (Figs. 5d, S7). On the other hand, cell proliferation was upregulated in the liver, which had insufficient tissue remodelling during the late regeneration phase (Fig. S5). Therefore, enhancing hepatocyte proliferation downregulated carbohydrate metabolism in the injured liver. In other words, liver injury changes the balance of nutritional demand for energy production associated with cell proliferation in the liver, and seems to reflect an optimal condition for liver regeneration. Interestingly, Huang et al. also reported that glucose supplementation decreases plasma amino acid levels, including BCAAs, during liver regeneration^38^. BCAAs regulate glucose metabolism through mTOR signalling pathways^30^; thus, BCAA and carbohydrate metabolism may mutually interact during liver regeneration after acute liver injury.

**Figure 6:**
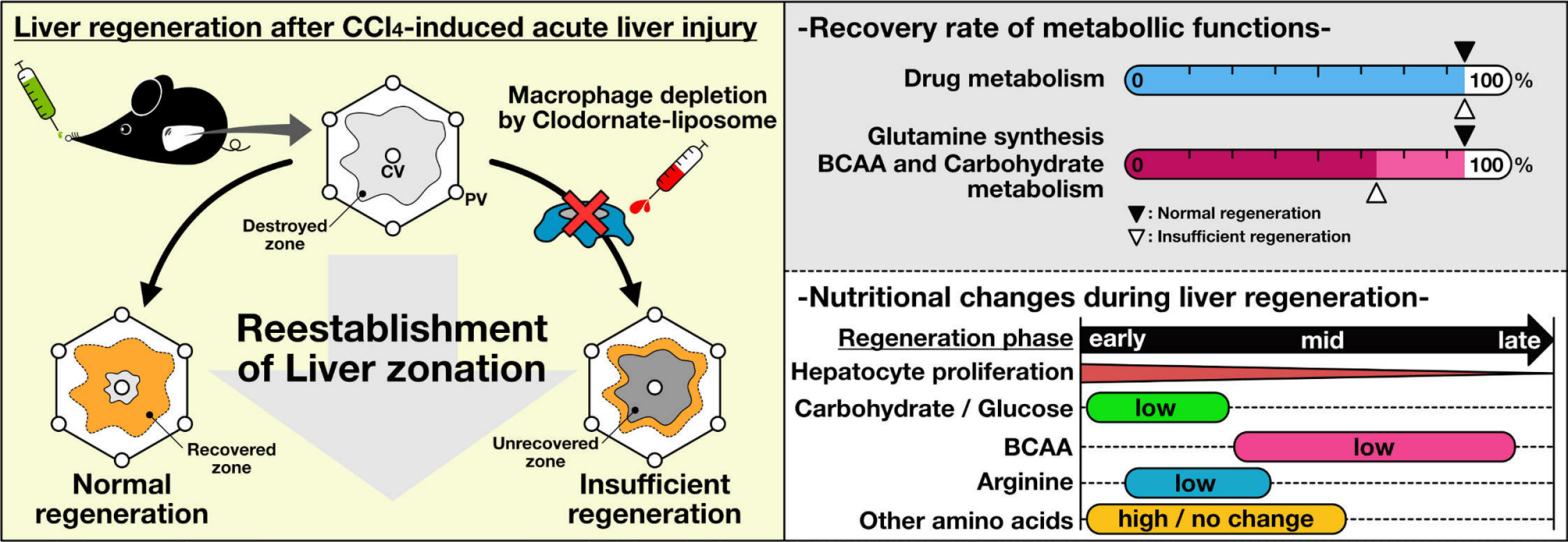
Overview of the study. Under normal conditions, liver macrophages remodel the injured liver tissues and promote recovery of liver metabolic functions during liver regeneration after acute liver injury. However, loss of macrophages causes insufficient tissue remodelling. Suppression of tissue remodelling is accompanied by defects in the recovery of amino acid and carbohydrate metabolism. However, drug metabolic functions retain regenerative capacity even if tissue remodelling is impaired.

In summary, we showed that liver macrophages repair injured tissue and promote the recovery of liver metabolic functions during liver regeneration after acute liver injury under normal tissue remodelling conditions (Fig. 6). By contrast, loss of macrophages, which suppressed tissue remodelling, caused insufficient recovery of amino acid, including glutamine and BCAA, and carbohydrate metabolism. Notably, we determined that drug metabolic functions retain their regenerative capacity even if tissue remodelling is impaired. We provide the first evidence for a link between macrophage-mediated tissue remodelling, and recovery of metabolic functions and liver zonation during liver regeneration. Furthermore, the findings increase our understanding of the machinery required for recovery of hepatic metabolism after acute liver injury. For the first time, this study shows the importance of hepatic metabolism during liver regeneration.

## Methods

### Acute liver injury and macrophage depletion mouse model

Male C57BL/6J mice (8–12 weeks old) were orally administered CCl_4_ (1.0 mL CCl_4_/kg body weight [b.w.]; × 4 diluted with olive oil) to induce acute liver injury after an overnight fast (n = 6/group). The total number of mice used in this study was 108 (48 mice for biochemical and metabolomic analyses; 48 mice for histological analyses; 12 mice for drug metabolism analyses). Approximately 48 h after CCl_4_-administration, the mice were injected intraperitoneally with CLOs (100 or 200 µL; Clophosome-A, FormuMax Scientific Inc., Sunnyvale, CA, USA), which specifically induces apoptosis of macrophages. Empty liposomes were used as the vehicle control. The liposomes were injected into mice every 48 h to maintain depleted macrophages. Liver and blood plasma samples were collected at 0, 48, 72, 96, and 144 h after CCl_4_ administration, and stored at − 80 °C until analyses. These mice were perfused with phosphate-buffered saline (PBS) followed by 4% paraformaldehyde (PFA) for histological analysis. We designed all experiments using laboratory animals based on the ARRIVE guidelines. The animal experiments were also performed following the National Institutes of Health Guide for the Care and Use of Laboratory Animals and were approved by the Nihon University Animal Care and Use Committee.

### Histological analysis

Haematoxylin–eosin (H&E) and Periodic acid Schiff (PAS) staining were performed according to standard protocols. DAB staining was used to detect Ki-67 in paraffin-embedded sections. Sliced tissues were stained with fluorophore-conjugated antibodies for immunofluorescent staining. Further methodological details of the Ki-67 and immunofluorescent staining, and TUNEL assay are described in the Supplementary Information.

### Immunoblotting

Frozen liver tissues (approx. 50 mg) were homogenised in five volumes of ice-cold homogenisation buffer (150 mM NaCl, 1 mM EDTA, 1% NP-40, 10 mM sodium fluoride, 1 mM sodium orthovanadate, and 1 mM β-glycerophosphate in 50 mM Tris–HCl, pH 7.8) supplemented with 0.5% protease inhibitor cocktail (P8340, Sigma-Aldrich, St. Louis, MO, USA). Homogenisation was performed using a TissueLyser LT (Qiagen, Valencia, CA, USA). After centrifugation (13,000×*g*, 15 min, 4 °C), the protein concentration of the tissue lysates was quantified with the BCA protein assay kit (Thermo Fisher Scientific, Waltham, MA, USA). Liver proteins were separated by electrophoresis (5 µg/well) and transferred to a polyvinylidene fluoride membrane. The membrane was blocked with 5% non-fat dry milk/PBS, and incubated with the following target-specific primary antibodies (1:2000) overnight at 4 °C: rabbit polyclonal anti-Cyp1a2 antibody (19936-1-AP, Proteintech, Rosemont, IL, USA ), rabbit polyclonal anti-Cyp2e1 antibody (AB1252, Millipore, Billerica, MA, USA), rabbit polyclonal anti-GS antibody (ab73593, Abcam, Cambridge, MA, USA), and goat polyclonal anti-Ass1 antibody (ab77590, Abcam). After three washes of 5 min with 0.05% Tween 20/Tris-buffered saline (TBS), the membrane was incubated with the appropriate secondary antibody for 30 min (1;20,000). The secondary antibodies used in this study were horseradish peroxidase (HRP)-conjugated goat anti-rabbit and rabbit anti-goat antibodies (Jackson Immuno Research, West Grove, PA, USA). Mouse monoclonal anti-β-actin antibody (1:5000; A5441, Sigma-Aldrich) and HRP-conjugated goat anti-mouse antibody (1:50,000; Jackson Immuno Research) were used as loading controls. The primary antibodies were diluted with Can Get Signal Immunoreaction Enhancer Solution 1 (Toyobo, Osaka, Japan). The secondary antibodies were diluted with 1% non-fat dry milk/PBS. The membranes were washed three times for 5 min and developed with Immunostar LD (Fujifilm Wako Pure Chemical, Osaka, Japan). Chemiluminescence was captured by the ChemiDoc MP imaging system (Bio-Rad, Hercules, CA, USA). To avoid overexposure, the exposure time was manually selected so that the chemiluminescent signal was within the dynamic range of the imaging system. The image obtained from the blot was not received any modification. Densitometric analysis was performed by using imageJ/Fiji (https://imagej.net/).

### qPCR

Liver samples were homogenised with TissueLyser LT (Qiagen). Total liver RNA was extracted using RNAiso plus (TaKaRa Bio, Shiga, Japan), and further purified according to the manufacturer’s protocol. The total RNA concentration of the purified extracts was quantified by NanoDrop Lite (Thermo Fisher Scientific). RNA quality was assessed by agarose gel electrophoresis. Total RNA was reverse-transcribed to cDNA using the PrimeScript RT reagent Kit (TaKaRa Bio). To analyse mRNA expression, cDNA was mixed with KOD SYBR qPCR Mix (Toyobo), and measured using the StepOne Realtime PCR System (Thermo Fisher Scientific). Gene expression levels were calculated by the delta-delta-Ct method. Rn18s (18S rRNA) was used as the internal standard. The primer pairs are listed in Supplementary Table S1.

### In vivo drug metabolism analysis

A drug metabolism analysis was performed according to previous studies with minor modifications^40,41^. Phenacetin (Sigma-Aldrich) and chlorzoxazone (Sigma-Aldrich) were mixed in a vehicle solution (5% DMSO, 5% EtOH, 15% water, 35% PEG200, and 40% PBS) to prepare the probe drug cocktail. The drug cocktail was given orally to mice (5 mg/kg b.w.; 10 mL/kg b.w.), and whole blood was repeatedly collected from these mice via the tail vein at 15, 30, 45, 60, 120, 240, 360, and 480 min. Blood plasma was collected by centrifugation and stored at − 80 °C until analysis. Liquid chromatography-tandem mass spectrometry (LC/MS/MS) was used to quantify the plasma drug concentration. Blood plasma (10 µL) was mixed with 400 µL acetonitrile (ACN) supplemented with internal standards (IS; 5 nM harmaline and 5 nM warfarin) for deproteinisation. To prepare the calibration curve, a 10 µL aliquot of a standard mixture of phenacetin and chlorzoxazone was mixed with 400 µL ACN (containing IS). After centrifugation (20,000×*g*, 15 min, 4 °C), the supernatant was dried in a centrifugal concentrator for 1 h. The precipitates were redissolved in 20 µL of 20% MeOH and centrifuged at 20,000×*g*. The supernatants were subjected to LC/MS/MS. Reverse-phase chromatography was performed on an Acquity UPLC system equipped with an InertSustain C18 column (150 mm × 2.1 mm ID, 3 µm particle size, GL Science, Tokyo, Japan). Mobile phase A was 0.1% formic acid (FA)/water and mobile phase B was 0.1% FA/ACN. The analytes were separated in gradient mode: 2% B for 0–0.5 min, 40% B at 2 min, 95% B at 2.5–5 min, and 2% B at 5.01–7.5 min. The column temperature was maintained at 60 °C, flow rate was set to 0.2 mL/min, and injection volume was 7.5 µL. The analytes were detected on a Quattro Premier XE system (Waters Corp., Milford, MA, USA) in ESI positive/negative mode. The MRM transition setting of each drug was as follows: phenacetin (180.0 > 110.1; ESI +), harmaline (215.2 > 172.1; ESI +), chlorzoxazone (167.8 > 131.9; ESI−), and warfarin (306.8 > 160.9; ESI−). Raw data were processed with MassLynx software (Waters), and the pharmacokinetic parameter (area under the concentration–time curve from zero to infinity [AUC_0–∞_]) was calculated by non-compartmental analysis using the PKNCA package in R software (R Foundation for Statistical Computing, Vienna, Austria)^42^.

### GC/MS-based untargeted metabolomics

A liver metabolomic analysis was performed by GC/MS. The data analysis was performed using MS-DIAL 3.70^43^ and R software. Pathway analysis was performed using MetaboAnalyst 4.0^44^. Further methodological details of the untargeted metabolomics analysis are described in Supplementary Information.

### Plasma amino acid profiling

The amino acid concentrations in blood plasma were quantified by the LC/MS/MS system used for drug quantification. Derivatisation and analysis were performed with the AccQ-Tag Ultra Derivatization Kit and an AccQ-Tag Ultra RP column (100 mm, 2.1 mm ID, 1.7 µm particle size; Waters). The amino acid standards were solubilised in 0.1 N HCl and diluted to prepare the calibration curve. Norvaline was used as the internal standard. Data analysis was performed using MassLynx software (Waters), and subsequent data processing was performed in R software.

### Plasma biochemical analysis

The alanine aminotransferase (ALT) and aspartate aminotransferase (AST) activity, and ammonia levels in blood plasma were measured by a chemical-analyser (DRI-CHEM 4000V, FUJIFILM, Tokyo, Japan).

### Statistical analysis

Statistical analysis was performed using Microsoft Excel (Microsoft Inc., Redmond, WA, USA). As the intergroup variances in mice with liver injury were unequal, Welch’s (two-tailed unpaired and unequal variances) *t*-test was used for two-group comparisons, and p-values < 0.05 were considered significant. R software was used for the statistical analysis of all pharmacokinetic parameters. Exploratory data analyses were also performed in R software using the analytical packages.

## Supplementary Information

Supplementary Information.

## Data Availability

The datasets generated or analysed in the current study are available from the corresponding author upon reasonable request.
