# Supplementary material for: Macrophage potentiates the recovery of liver zonation and metabolic function after acute liver injury
Source: Sci Rep. 2021 May 6;11:9730. doi: 10.1038/s41598-021-88989-9 (PMC8102573; doi:10.1038/s41598-021-88989-9)

Supplementary Information

Macrophage potentiates the recovery of liver zonation and metabolic function after acute liver injury remodelling during liver regeneration

Atsushi Miura[1], Takashi Hosono[2,3], Taiichiro Seki[1,2,3,*]

[1] General Research Institute, Nihon University Collage of Bioresource Sciences, Fujisawa, Kanagawa 252-0880, Japan
[2] Department of Applied Life Sciences, Nihon University Graduate School of Bioresource Sciences, Fujisawa, Kanagawa 252-0880, Japan
[3] Department of Chemistry and Life Science, Nihon University Collage of Bioresource Sciences, Fujisawa, Kanagawa 252-0880, Japan
*Corresponding author: seki.taiichirou@nihon-u.ac.jp

Index

Supplementary Table 2
Supplementary Methods 3
Supplementary Figures 7
Raw blot images 14

Supplementary Table S1. Primer-paires

Gene symbol	Forward	Reverse
***Adgre1* (F4/80)**	CCCCAGTGTCCTTACAGAGTG	GTGCCCAGAGTGGATGTCT
Cd68	CCATCCTTCACGATGACACCT	GGCAGGGTTATGAGTGACAGTT
Clec4f	CTTCGGGGAAGCAACAACTC	CAAGCAACTGCACCAGAGAAC
CD11b	ATGGACGCTGATGGCAATACC	TCCCCATTCACGTCTCCCA
Ly6c	GCAGTGCTACGAGTGCTATGG	ACTGACGGGTCTTTAGTTTCCTT
Ccr2	ATCCACGGCATACTATCAACATC	CAAGGCTCACCATCATCGTAG
Cx3cr1	GAGTATGACGATTCTGCTGAGG	CAGACCGAACGTGAAGACGAG
Ccnd1	ACCCTGACACCAATCTCCTCAAC	ACGATTTTCCGCATGGATGGC
Ccne1	CTCGGGTGTTGTAGGTTGCT	CTGTTGGCTGACAGTGGAGA
Ccna2	TGCAGCCTGCAAACTGTAAGG	GCAGCTCCAGCAATGAGTGAA
Ccnb1	AATCCTTGCAGTGAGTGACG	CCAGTTGTCGGAGATAAGCA
Tnfa	GAGAAAGTCAACCTCCTCTCTG	GAAGACTCCTCCCAGGTATATG
Il6	GCAAGAGACTTCCATCCAGTTGC	AAGTCTCCTCTCCGGACTTGTG
Rn18s	AGTTCCGACCATAAACGATGCC	AAGACTTTGGTTTCCCGGAAGC

Supplementary Methods

Histological analysis

PFA perfusion-fixed liver tissues were additionally post-fixed with 4% PFA for 24 h, dehydrated, and embedded in paraffin. Paraffin-embedded tissues were sectioned by a microtome at 5 μm thickness. Tissue sections were incubated in 10 mM sodium citrate buffer (pH 6.0) at 110°C for 20 min to retrieve the antigen for Ki-67 immunostaining. The tissue sections were incubated in 3% H_2O_2/methanol (MeOH) for 10 min to quench endogenous peroxidase activity. The tissue sections were washed with PBS, blocked in 5% bovine serum albumin (BSA)/PBS for 40 min, and incubated with rabbit monoclonal anti-Ki67 [SP8] antibody (1:40 in 0.5% BSA/PBS; GTX16667, Gene Tex) at 4°C overnight, followed by washes with PBS and incubation with peroxidase-conjugated Affinipure goat anti-rabbit IgG (H+L) (1:1000 in 0.5% BSA/PBS; Jackson Immuno Research) for 2 h. The Ki-67-positive signal was detected with the ImmPACT DAB Peroxidase Substrate Kit (SK-4105, Vector Laboratories). Histological images were captured by the ZEISS Axio Imager A2 upright microscope.

Three different images were collected from one tissue section in each mouse to quantify the number of Ki-67 positive nuclei, the PAS positive area and the necrotic cell area. Images were quantified using the ImageJ macro in Fiji software.

For immunofluorescent staining, post-fixed liver tissues were sliced at 50 μm thickness with a microslicer (Neo-LinearSlicer MT, Dosaka EM Co. Ltd.). The tissue slices were washed twice with PBS-TX100 (0.2% w/v Triton X-100 and 0.05% w/v sodium azide in PBS) for 5 min. The tissue slices were sequentially incubated in three permeabilisation buffers as follows: Buffer A (0.2% w/v Triton X-100, 20% v/v DMSO, and 0.05% w/v sodium azide in PBS); Buffer B (0.1% w/v Tween-20, 0.1% w/v Triton X-100, 0.1% w/v deoxycholic acid, 0.1% w/v NP-40, 20% v/v DMSO, and 0.05% w/v sodium azide in PBS); and Buffer C (0.2% w/v Triton X-100, 0.3 M glycine, 20% v/v DMSO, and 0.05% w/v sodium azide in PBS). Each permeabilisation step was performed at 37°C for 30 min. The tissue slices were blocked in blocking buffer (0.2% w/v Triton X-100, 6% donkey serum, and 10% v/v DMSO in PBS) for 1 h at 37°C. Rabbit polyclonal anti-Cyp1a2 antibody (1:300;

19936-1-AP, Proteintech), rabbit polyclonal anti-Cyp2e1 antibody (1:400; AB1252, Millipore), rabbit polyclonal anti-GS antibody (400:1; ab73593, Abcam), rat monoclonal anti-F4/80 [CI:A3-1] (1:300; ab6640, Abcam), rabbit polyclonal anti-fibrin antibody (1:400; Dako), and goat polyclonal anti-Ass1 antibody (1:200; ab77590, Abcam) were incubated for 24 h at 37°C. The tissue slices were washed four times with wash buffer (0.2% w/v Tween 20, 10 μg/mL heparin, and 0.05% w/v sodium azide in PBS) for 10 min each and incubated with the appropriate secondary antibody (1:1000) and DAPI (1:2000) for 20 h at 37°C. The secondary antibodies used were as follows: donkey anti-rabbit IgG (H+L) AlexaFluor 488 conjugated antibody (A-21206, Thermo Fisher Scientific), donkey F(ab')2 anti-rat IgG H&L AlexaFluor 647 conjugated antibody (ab150151, Abcam), and donkey F(ab')2 anti-goat IgG H&L AlexaFluor 555 conjugated antibody (ab150138, Abcam). The primary and secondary antibodies were diluted in antibody buffer (0.2% w/v Tween 20, 10 μg/mL heparin, 3% v/v donkey serum, 5% v/v DMSO, and 0.05% w/v sodium azide in PBS). The tissue slices were washed four times with wash buffer for 10 min each and mounted with FluorSave Reagent (345789, Millipore). Fluorescent images were captured by a Leica SP8 confocal microscope. The pseudo-colour images (R, G, and B) were merged to the RGB image using Fiji software. Combined images were arranged by Affinity Designer software (Serif, UK, https://affinity.serif.com/).

Three different images were collected from one tissue section in each mouse to quantify the fibrin positive area and the F4/80 positive cells. Images were quantified using the ImageJ macro in Fiji software.

Apoptotic cells detection (TUNEL assay) was performed by using In Situ Cell Death Detection Kit Fluorescein (11684795910, Roche) according to manufacturer's instruction. Briefly, liver slices were peremeabilised with 0.5%v/v Triton X-100 in PBS for 1 h at 37°C. The tissue slices were incubated with a reaction enzyme mixture for 2 h at 37°C and mounted with FluorSave Reagent. Images were captured by a Leica STELLARIS 5 confocal microscope. Three different images were collected from one tissue section in each mouse to quantify the TUNEL positive nuclei. Images were quantified using the ImageJ macro in Fiji software.

GC/MS-based untargeted metabolomics

Frozen liver tissues (50 mg) were homogenised in 1 mL solvent mixture (MeOH/chloroform/water = 2.5:1:1 v/v/v). Then, 10 µL of an internal standard solution (1.2 mg/mL ribitol in water) was added to the homogenates, and the homogenates were vigorously mixed on a temperature-controlled shaker (MBR-022UP, Taitec) for 30 min at 20°C. Subsequently, the homogenates were centrifuged at 16,000 × *g* for 5 min at 4°C to discard insoluble matter. Water (200 µL) was added to the supernatants and mixed well. These mixtures were centrifuged (20,000 × *g*, 15 min, 4°C) to separate the liquid phase. The hydrophilic phase of the centrifuged supernatants (600 µL of the upper layer) was transferred to new tubes. The upper layer of every sample was mixed in a single tube, and dispensed to new tubes (600 µL/tube) to make pooled quality control (QC) samples. The extracted samples and pooled QCs were snap-frozen in liquid nitrogen and dried in a centrifugal concentrator overnight. To prevent technical/instrumental biases, the samples were randomised using the randomise (RAND) function in Microsoft Excel. The pooled QCs were analysed at six sample intervals (*i.e.*, six samples and one QC were grouped into one batch). Derivatisation steps were performed for every batch. Prior to TMS derivatisation, 100 µL of 20 mg/mL methoxyamine hydrochloride (MeOX)/pyridine was added to the dried samples, and the resolved samples were sonicated twice for 20 s each in an ice bath. The MeOX mixed samples were incubated in a temperature-controlled shaker for 90 min at 30°C. Then, 50 µL of *N*-methyl-*N*-trimethylsilyl-trifluoroacetamide (MSTFA; Sigma-Aldrich) was added to the MeOX mixed samples, and the MSTFA mixed samples were incubated in a temperature-controlled shaker for 30 min at 30°C. The pooled QCs were derivatised using the same procedure. Analysis of the derivatised samples was performed by gas chromatography/mass spectrometry (GC/MS). An analytical system was constructed with the Agilent 7890B GC and a single JEOL JMS-Q1500GC (electro ionisation-single quadrupole). The derivatised samples (1 µL) were injected into the GC at a 25:1 split ratio, and the analytes were separated on a InertCap 5MS/NP column (30 m, 250 µm ID, 0.25 µm film thickness; GL Science), using helium as the carrier gas (constant flow of 1.120 mL/min). Column oven temperature was held at 80°C for 2 min, increased to 330°C (15°C/min), and held for 9 min. Other

analytical conditions were as follows: injector temperature, 250°C; septum purge flow, 5 mL/min; solvent delay, 3.5 min; MS ion source temperature, 200°C; MS interface temperature, 250°C; and MS scan mode, 85–500 m/z scan. A saturated hydrocarbon standard mixture (1021-58321, GL Science) was used to adjust retention time drifts. A retention index (RI) was calculated from retention time of the alkanes. Raw experimental data were converted to netCDF type data using JEOL Escrime software. Then, netCDF data were converted to Abf type data on the Reifycs Abf Converter. Spectral deconvolution and peak identification were performed using MS-DIAL 3.70 software coupled with a reference database (GL-Science DB Kovats RI; for InertCap 5MS/NP column) downloaded from the RIKEN PRIMe website (http://prime.psc.riken.jp/). Normalised peak intensity of the metabolites in each sample was calculated using MS-DIAL software. Exported raw text data from MS-DIAL software were converted into a data table format using R scripts written in-house.

The peak intensity of the metabolites was standardised to a mean of 0 and variance of 1 prior to the data analysis. Principal component analysis, Pearson's correlation coefficient calculation, hierarchical clustering, and data visualisation were performed in R software. The pathway analysis was performed at the Metaboanalyst 4.0 website (https://www.metaboanalyst.ca/).

Supplementary Figures

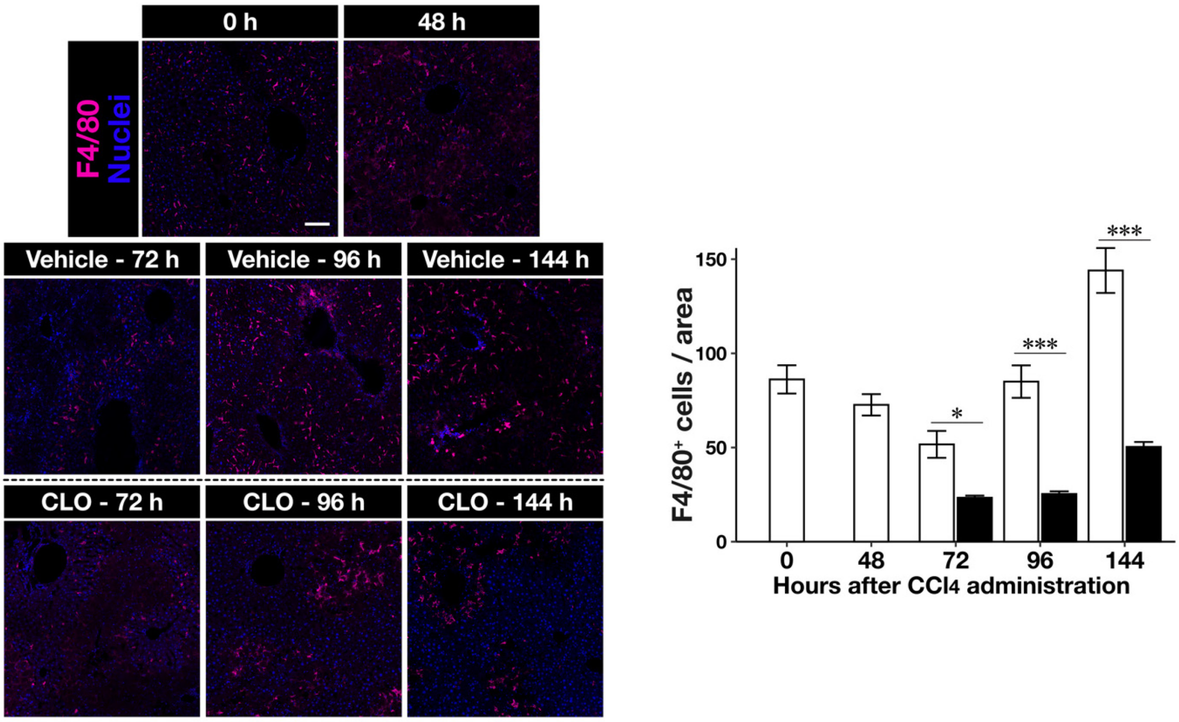

Supplementary Figure S1. The effect of clodronate-loaded liposome (CLO) treatment on F4/80 expression in the mouse liver.

Representative images of F4/80 immunofluorescent stained liver sections (scale bar, 100 µm); magenta, F4/80; blue, cell nuclei. Image quantification was performed by using image J. Data are presented as mean ± SEM (n = 6/group). Intergroup differences between the vehicle and CLO group at each time-point were compared using Welch's *t*-test; $^{*}p < 0.05$, $^{***}p < 0.001$.

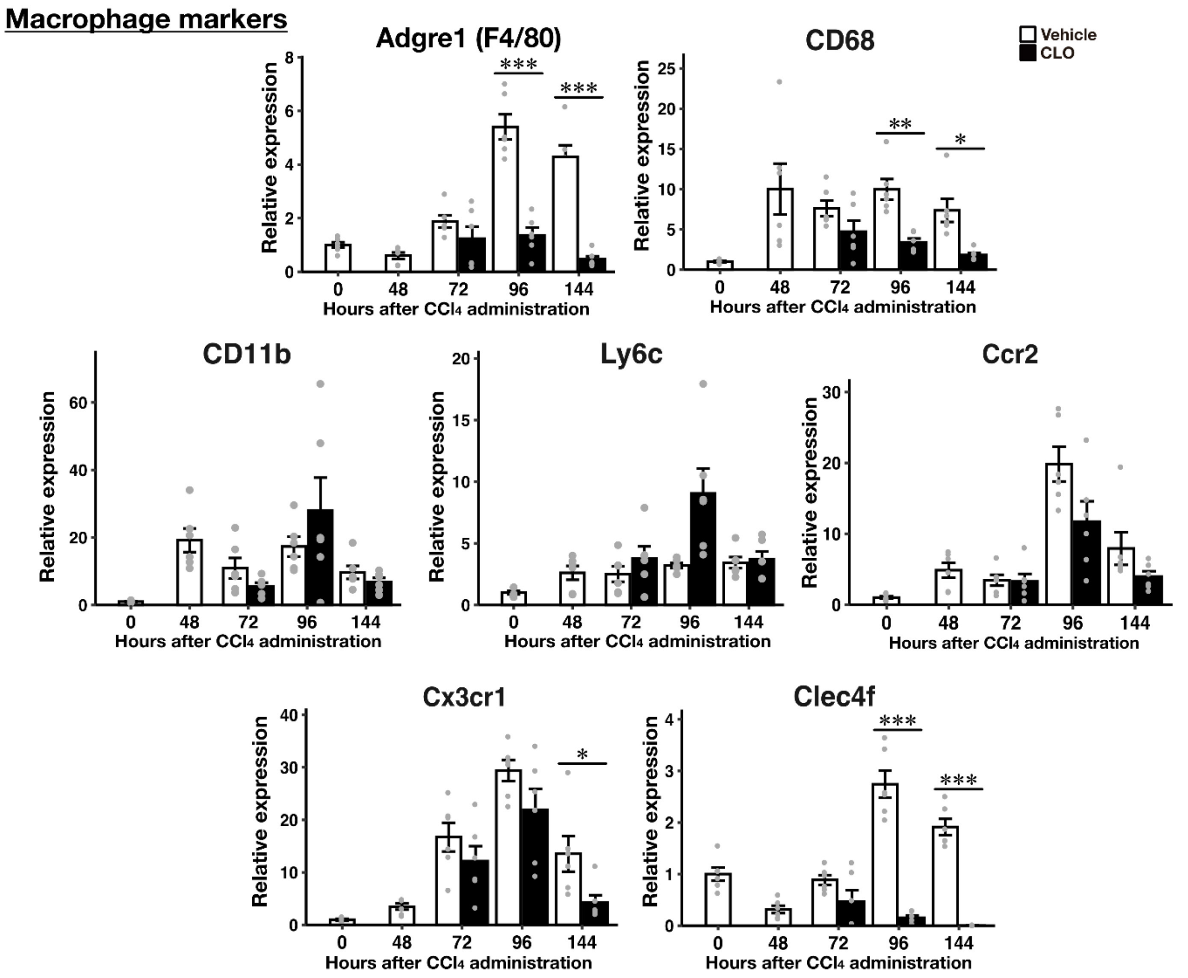

Supplementary Figure S2. The effect of clodronate-loaded liposome (CLO) treatment on expression of macrophage marker genes in the mouse liver.

Gene expression levels in the liver were measured by qPCR and normalised with 18S rRNA. White bars indicate vehicle-treated mice, and black bars indicate CLO-treated mice. Data are presented as mean ± standard error of the mean (SEM; n = 6/group). Intergroup differences between the vehicle and CLO group at each time-point were compared using Welch's *t*-test; *$p < 0.05$, **$p < 0.01$, ***$p < 0.001$. Adgre1, adhesion G protein-coupled receptor E1; Clec4f, C-type lectin domain family 4 member F.

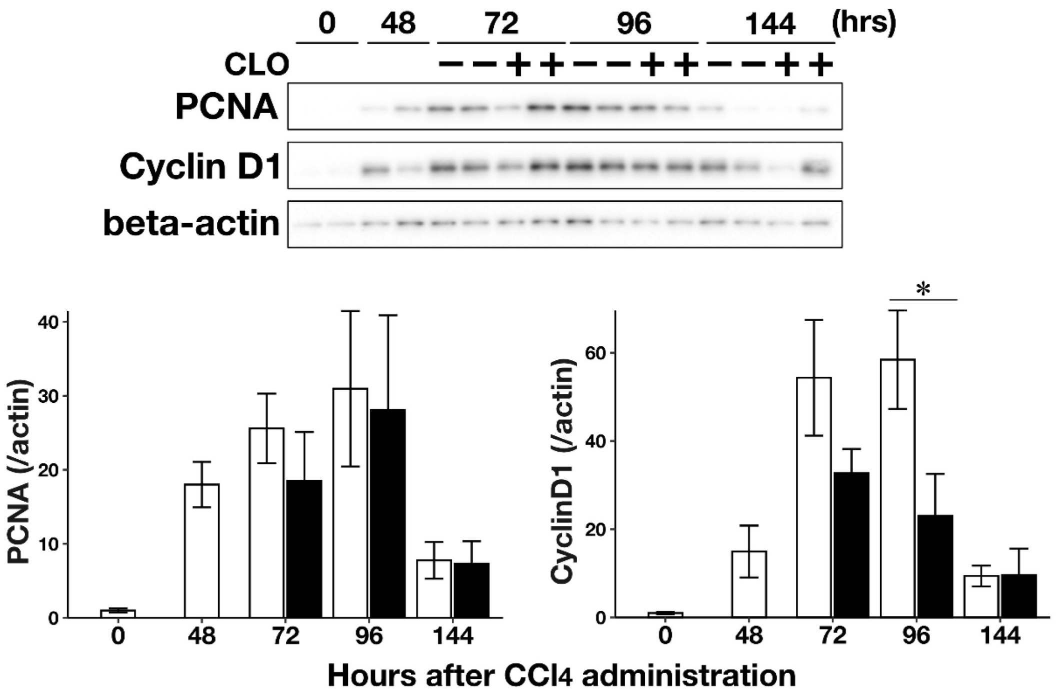

Supplementary Figure S3. The effect of macrophage depletion on protein expression of PCNA and cyclin D1 in the mouse liver.

Proliferating cell nuclear antigen (PCNA) and cyclin D1 protein expression measured using immunoblotting. β-actin was used as loading controls. Data are presented as mean ± SEM (n = 6/group). Intergroup differences between the vehicle and CLO group at each time-point were compared using Welch's *t*-test; $^*p < 0.05$.

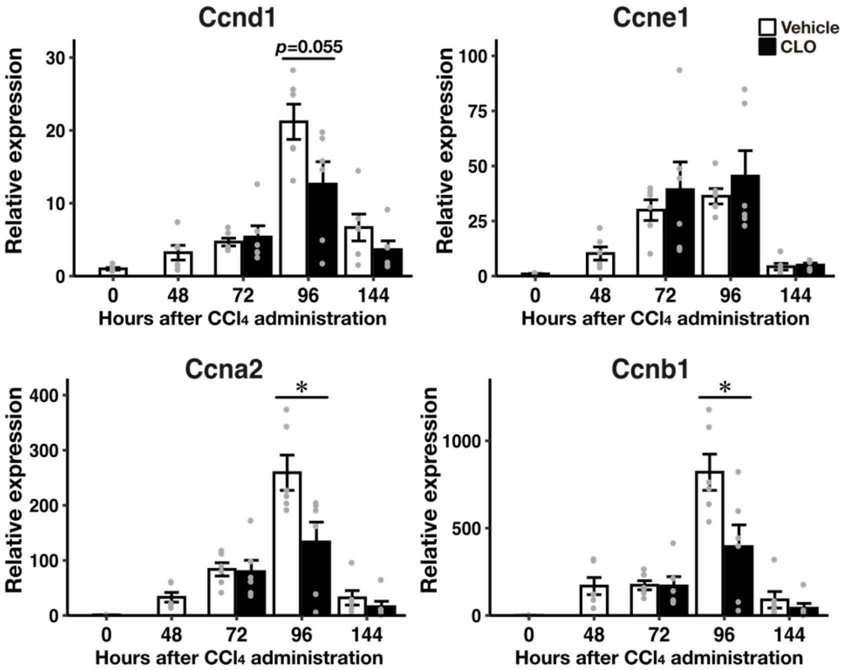

Supplementary Figure S4. The effect of macrophage depletion on expression of cyclins in the mouse liver.

Gene expression levels of cell cycle genes were measured by qPCR and normalised with 18S rRNA. White bars indicate vehicle-treated mice and black bars indicate CLO-treated mice. Data are presented as mean ± SEM (n = 6/group). Intergroup differences between the vehicle and CLO group at each time-point were compared using Welch's *t*-test; *p < 0.05. Ccnd1, cyclin D1; Ccne1, cyclin E1; Ccna2, cyclin A2; Ccnb1, cyclin B1.

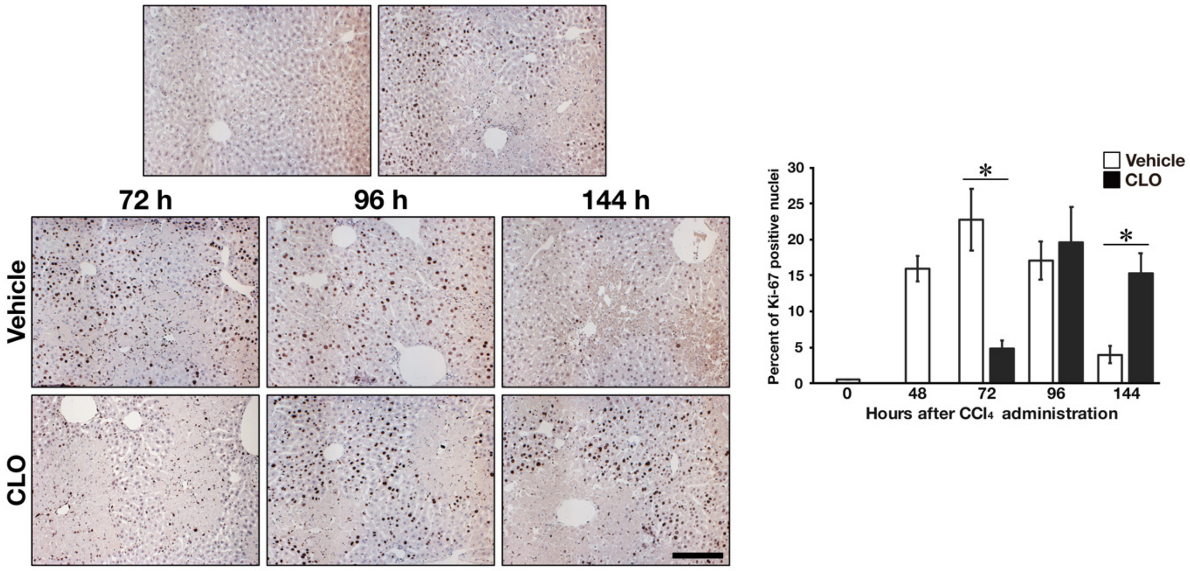

Supplementary Figure S5. The effect of macrophage depletion on Ki-67 expression in the mouse liver.

Representative images of Ki-67 immunohistochemistry (scale bar, 100 µm). Ki-67-positive nuclei are brown. The images were quantified with ImageJ software as follows: the number of brown dye-stained nuclei was divided by haematoxylin-stained (total) nuclei numbers counted from the same images. White bars indicate vehicle-treated mice and black bars indicate CLO-treated mice. Data are presented as mean ± SEM (n = 6/group). Intergroup differences between the vehicle and CLO group at each time-point were compared using Welch's *t*-test; *$p < 0.05$.

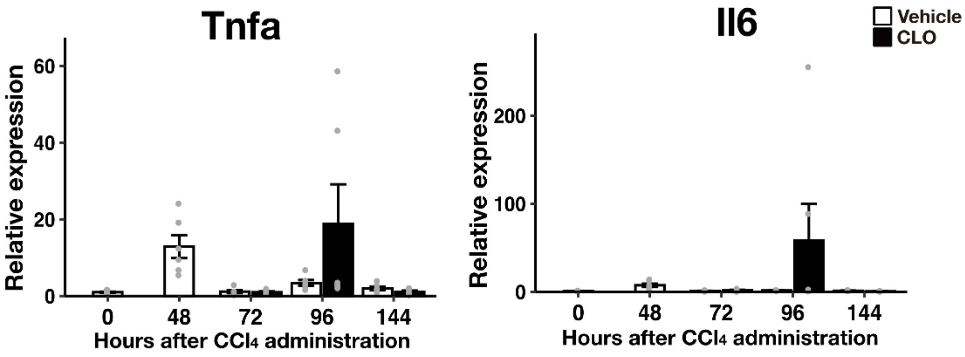

Supplementary Figure S6. The effect of macrophage depletion on gene expression of cell proliferative inflammatory cytokines in the mouse liver.

Gene expression levels of Tnfa and Il6 were measured by qPCR and normalised with 18S rRNA. White bars indicate vehicle-treated mice and black bars indicate CLO-treated mice. Data are presented as mean ± SEM (n = 6/group).

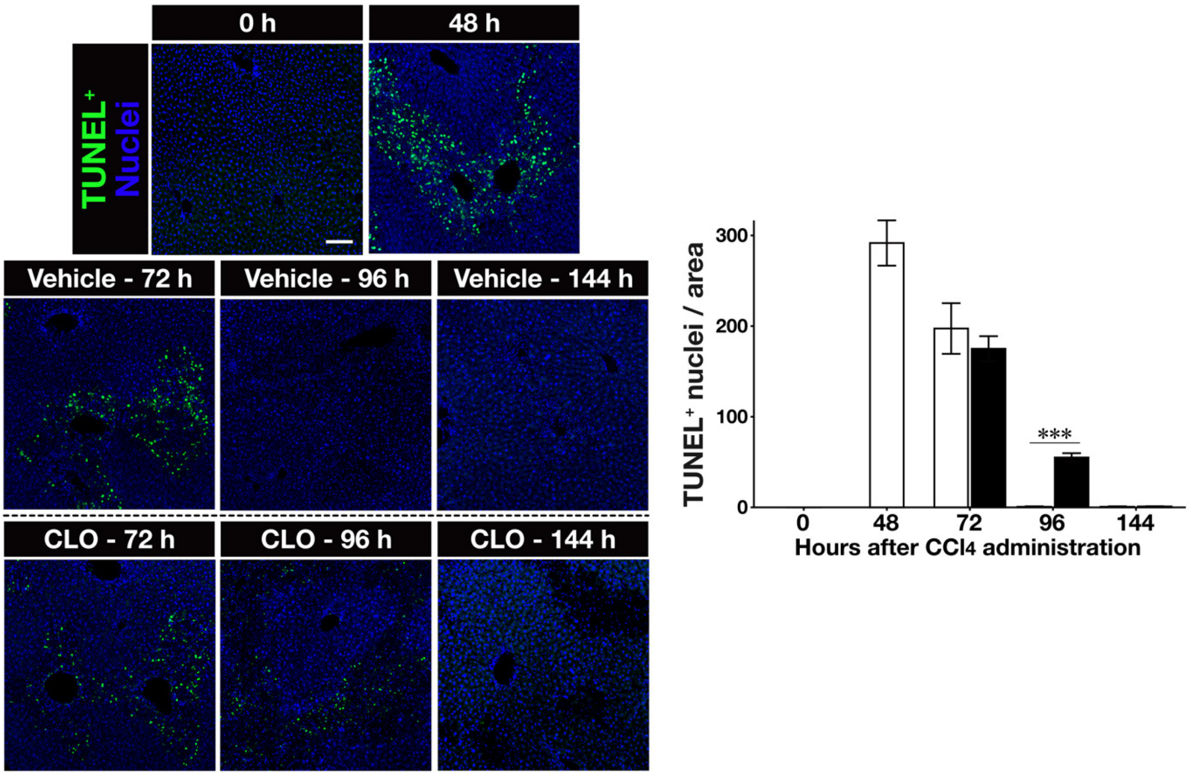

Supplementary Figure S7. The effect of macrophage depletion on apoptotic cell death in the mouse liver.

Representative images of apoptotic cells in liver sections (scale bar, 100 μm); green, TUNEL positive nuclei (apoptotic cells); blue, cell nuclei. Image quantification was performed by using image J. Data are presented as mean ± SEM (n = 6/group). Intergroup differences between the vehicle and CLO group at each time-point were compared using Welch's *t*-test; ***$p < 0.001$.

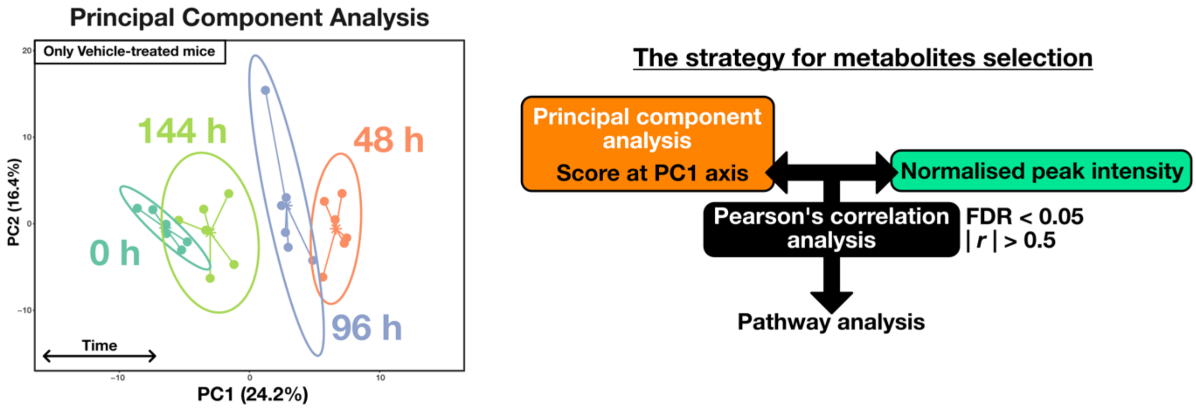

Supplementary Figure S8. Characterisation of the liver metabolome during regeneration after acute liver injury.
The score plot was obtained from the principal components analysis (PCA). Each point indicates an assay of one mouse. The ellipses denote the 95% confidence levels, and the asterisks located at the centre of each ellipse indicate the centroids of the ellipses. For pathway analysis, metabolites were filtered with statistical criteria calculated using Pearson's correlation analysis (correlation coefficient r > ± 0.5 and false discovery rate < 0.05).

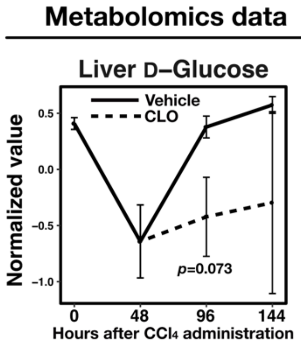

Supplementary Figure S9. Glucose levels in the regenerating liver after acute liver injury.
Liver glucose levels obtained from our metabolomic data are shown. Data are presented as mean ± SEM (n = 6/group). Intergroup differences between the vehicle and CLO group at each time-point were compared using Welch's *t*-test; *p < 0.05.

Raw blot images

Figure 2b

Cyp1a2

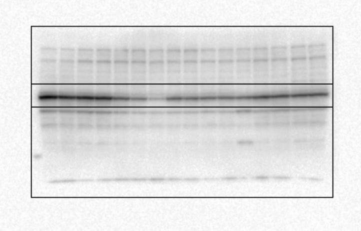

Cyp2e1

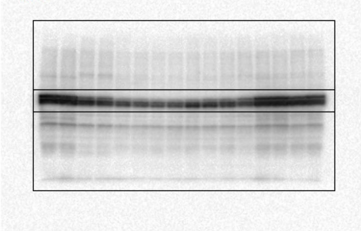

beta-actin

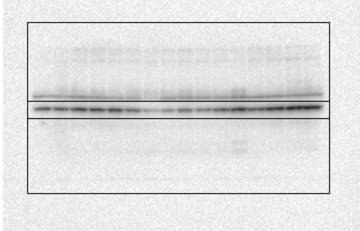

Figure 3c

GS

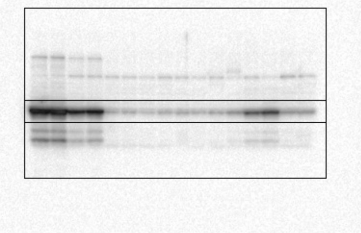

Ass1

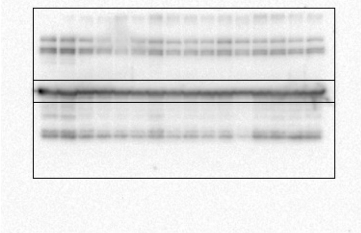

beta-actin

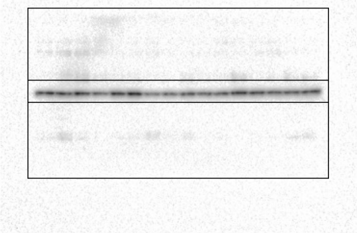

Supplementary figure 3

PCNA

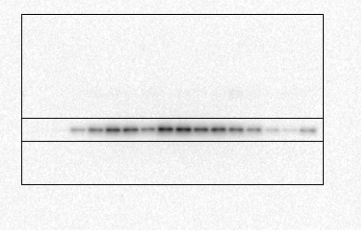

Cyclin D1

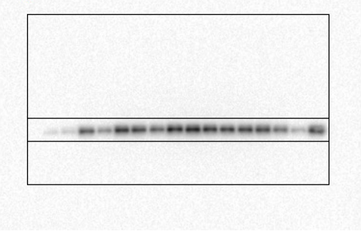

beta-actin

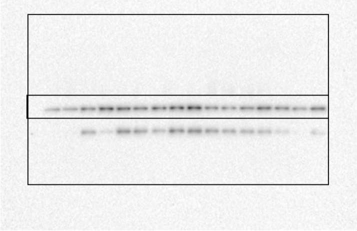

Supplement: Supplementary file 1 — Supplementary Information. [file 41598_2021_88989_MOESM1_ESM.pdf]
